# Exploring the Pharmacogenomic Map of Croatia: PGx Clustering of 522-Patient Cohort Based on UMAP + HDBSCAN Algorithm

**DOI:** 10.3390/ijms26020589

**Published:** 2025-01-12

**Authors:** Petar Brlek, Luka Bulić, Leo Mršić, Mateo Sokač, Eva Brenner, Vid Matišić, Andrea Skelin, Lidija Bach-Rojecky, Dragan Primorac

**Affiliations:** 1 St. Catherine Specialty Hospital, 10000 Zagreb, Croatia; luka.bulic0302@gmail.com (L.B.);; 2 School of Medicine, Josip Juraj Strossmayer University of Osijek, 31000 Osijek, Croatia; 3 Faculty of Science, Department of Molecular Biology, University of Zagreb, 10000 Zagreb, Croatia; 4 Department of Information Systems and Business Analytics, Algebra University, 10000 Zagreb, Croatia; 5 Genos Glycoscience Research Laboratory, 10000 Zagreb, Croatia; 6 Faculty of Pharmacy and Biochemistry, University of Zagreb, 10000 Zagreb, Croatia; 7 Eberly College of Science, The Pennsylvania State University, State College, PA 16802, USA; 8 School of Medicine, University of Split, 21000 Split, Croatia; 9 The Henry C. Lee College of Criminal Justice and Forensic Sciences, University of New Haven, New Haven, CT 06516, USA; 10 Regiomed Kliniken, 96450 Coburg, Germany; 11 School of Medicine, University of Rijeka, 51000 Rijeka, Croatia; 12 Faculty of Dental Medicine and Health, Josip Juraj Strossmayer University of Osijek, 31000 Osijek, Croatia; 13 School of Medicine, University of Mostar, 88000 Mostar, Bosnia and Herzegovina; 14 National Forensic Sciences University, Gandhinagar 382007, India

**Keywords:** adverse drug reactions, pharmacogenetics, unsupervised machine learning, hierarchical density-based clustering, dimensionality reduction

## Abstract

Pharmacogenetics is a branch of genomic medicine aiming to personalize drug prescription guidelines based on individual genetic information. This concept might lead to a reduction in adverse drug reactions, which place a heavy burden on individual patients’ health and the economy of the healthcare system. The aim of this study was to present insights gained from the pharmacogenetics-based clustering of over 500 patients from the Croatian population. The data used in this article were obtained by the pharmacogenetic testing of 522 patients from the Croatian population. The patients were clustered based on the genotypes of 28 pharmacologically relevant genes. Dimensionality reduction was employed using the UMAP algorithm, after which clusters were defined using HDBSCAN. Validation of clustering was performed by decision tree analysis and predictive modeling using the RandomForest, XGBoost, and ExtraTrees classification algorithms. The clustering algorithm defined six clusters of patients based on two UMAP components (silhouette score = 0.782). Decision tree analysis demonstrated *CYP2D6* and *SLCO1B1* genotypes as the main points of cluster determination. Predictive modeling demonstrated an excellent ability to discern the cluster of each patient based on all genes (avg. ROC-AUC = 0.998), *CYP2D6* and *SLCO1B1* (avg. ROC-AUC = 1.000), and *CYP2D6* alone (avg. ROC-AUC = 0.910). Membership in each cluster provided clinically relevant information, in the context of ruling out certain favorable or unfavorable phenotypes. However, this study’s main limitation is its cohort size. Through further research and investigation of a larger number of patients, more accurate and clinically applicable associations between pharmacogenetic genotypes and phenotypes might be discovered.

## 1. Introduction

An adverse drug reaction (ADR) implies an undesired and potentially harmful response to pharmacological treatment. According to the literature, primary care reports show an 8–20% pooled prevalence of ADRs, depending on the observed studies [1]. According to data from The Agency for Medicinal Products and Medical Devices of Croatia (HALMED), almost 10,000 ADRs were reported in Croatia in 2021, which was almost 50% more than the previous year [2]. ADRs represent a great issue, not only for individuals but also for the entire healthcare system. An analysis by Sultana et al. (2013) presented the burden of ADRs from both a clinical and economic aspect [3]. For this reason, new branches of modern medicine has endeavored to explore the potential of personalized pharmacotherapy based on individual genetic data.

A branch of genomic medicine focused on investigating how specific genotypes of certain genes can affect the drug response is pharmacogenetics (PGx). With the rapid technological advancements in genomic medicine, PGx is becoming more and more integrated into clinical practices by the day [4]. A significant number of genotype–phenotype correlations have already been discovered, providing the necessary knowledge for drug dose adjustment and ADR avoidance [5]. A study by Matišić et al. (2022) demonstrated that actionable drug–gene pairs could be found in 74% of 319 included patients from the Croatian population [6]. This finding stresses the importance of considering PGx results when prescribing pharmacotherapy. Data relevant in this context have also been found on a much larger scale. through long-term studies demonstrating drug serum levels inconsistent with the therapeutic standards in a significant proportion of patients [7].

Machine learning (ML) has become a powerful tool in data analysis and has recently found its application in many fields of research, one of which is certainly biomedicine [8]. Through its robust ways of data analysis, ML has the potential to provide novel conclusions that could be reached through traditional data analysis. ML has several subcategories which deal with different types of problems. Supervised machine learning implies solving tasks such as the input-based value prediction of categorical (classification) or continuous (regression) variables based on previously known input–output pairs. On the other hand, unsupervised learning implies tasks such as input data clustering, which produces a new (previously unknown) categorical data descriptor [9]. Another useful data analysis technique in ML is dimensionality reduction, which reduces the number of descriptors a database has by combining them, simplifying any further analysis [10].

In this study, we employed dimensionality reduction and clustering algorithms to perform the clustering of 522 patients from the Croatian population, based on pharmacogenetic data. After validation of the clustering process with predictive modeling, specific pharmacogenetic information was extracted from the genotype distributions in each cluster. The aim of this process was to assess which additional insights into the individual and population pharmacogenetic profile can be gained and what clinical relevance they carry.

## 2. Results

### 2.1. Analysis of Clustering Results

#### 2.1.1. Descriptive Cluster Analysis

**Figure 1:**
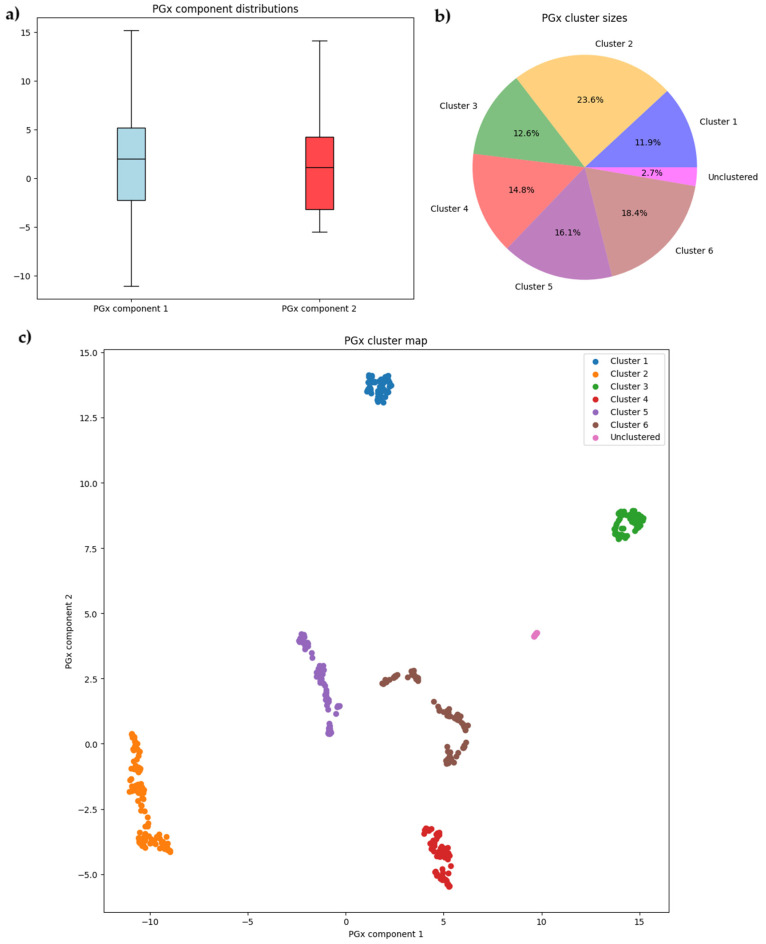
Detailed visual analysis of the clustering process, including reduced dimension component distributions (**a**), relative cluster sizes (**b**), and a visual representation of clusters in two dimensions (**c**).

The initial database consisted of 522 rows and 58 columns. Using the UMAP algorithm, the initial 58 dimensions were reduced to two multidimensional components. These components were defined as “PGx component 1” and “PGx component 2”. Distributions were determined for both components (Figure 1a). Based on the values of these two variables, the HDBSCAN algorithm identified six clusters of patients. Cluster sizes were equal to 62, 123, 66, 77, 84, and 96 for clusters 1–6, respectively. Only 14 patients were identified as noise and not clustered (2.7% of patients) and no unique genotypes were lost in this group (Figure 1b). The clusters are graphically presented based on their PGx component values (Figure 1c).

For this cluster distribution in two reduced dimensions, the silhouette score equaled 0.782, which was considered acceptable.

#### 2.1.2. Genotype Distribution Across Clusters

The presence of each genotype was determined for all six clusters (Supplement Table S1), and positively (present in only one cluster) and negatively specific (not present in only one cluster) genotypes were identified (Table 1).

A total of 64 positively specific genotypes were identified, 53 of which were identified in the *CYP2D6* gene (83%). The rest were found in the *CYP2B6, CYP3A4, CYP3A5, HLA-A, SLCO1B1,* and *VKORC1* genes. Additionally, 9 negatively specific genotypes were identified, distributed among the *CYP2C9, HLA-B, SLCO1B1,* and *F5* genes. The *CYP2C19, CYP2C-cluster, CYP4F2, COMT, DRD2, GRIK4, HTR2A, HTR2C, IFNL4, SLC6A4, F2,* and *MTHFR* genes were found to be completely non-specific with respect to genotype cluster distribution.

#### 2.1.3. Phenotype Distribution Across Clusters

The presence of each phenotype was determined across all six clusters (Supplement Table S2), and positively (present in only one cluster) and negatively specific (not present in only one cluster) phenotypes were identified (Table 2).

A total of 5 positively specific phenotypes were identified, distributed among the *CYP2B6, CYP3A4, CYP3A5, HLA-A,* and *VKORC1* genes. Additionally, 7 negatively specific phenotypes were identified, distributed among the *CYP2D6, HLA-B, SLCO1B1,* and *F5* genes. The *TPMT* and *UGT1A1* genes were found to be completely non-specific with respect to phenotype cluster distribution, on top of all the genes identified as genotype cluster-non-specific in the previous step of analysis.

### 2.2. Analysis of Clustering Validation

**Figure 2:**
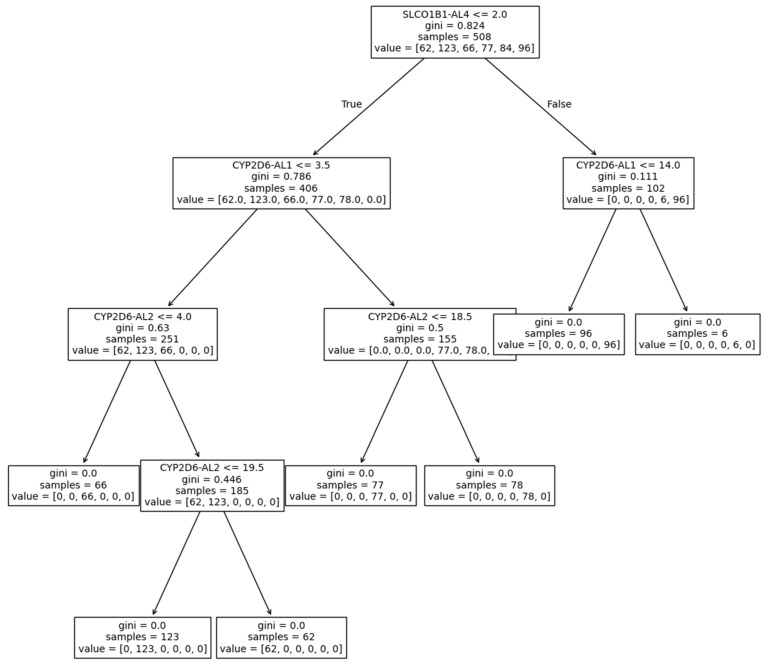
Decision tree for cluster determination based on DecisionTreeClassifier model (the gini value refers to the rate of incorrect labeling based on the sample distribution, for the samples at that level of the tree).

A DecisionTreeClassifier model was fitted to the data and cluster labels in order to produce a visually interpretable decision tree (Figure 2).

The tree demonstrated that cluster determination was performed based on *CYP2D6* and *SLCO1B1* alleles. Based on these findings, more robust prediction models were evaluated based on all genes, only *CYP2D6* and *SLCO1B1* alleles, only *CYP2D6* alleles, and only *SLCO1B1* alleles. Accuracies, F1 scores, and ROC-AUC scores were determined as quality metrics (Table 3).

**Figure 3:**
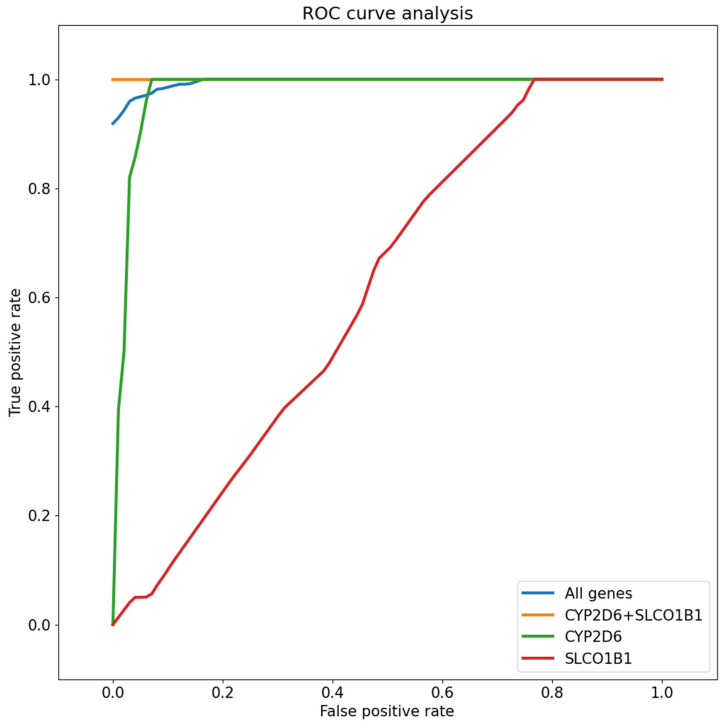
ROC curves (one-versus-rest) for cluster prediction based on all genes, *CYP2D6* and *SLCO1B1* alleles, *CYP2D6* alleles, and *SLCO1B1* alleles.

Additionally, ROC curves were plotted for each instance of testing, as well as the average (Figure 3).

The validation results demonstrate a strong ability to infer which cluster the patient belongs to from their pharmacogenetic data (avg. ROC-AUC = 0.998). The highest results were achieved when only *CYP2D6* and *SLCO1B1* alleles were observed (avg. ROC-AUC = 1.000). The quality metrics remained high when only *CYP2D6* alleles were used for cluster prediction (avg. ROC-AUC = 0.910). When observing predictive power based on *SLCO1B1* alleles alone, the quality of the predictions declined drastically (avg. ROC-AUC = 0.662).

## 3. Discussion

Using a complex clustering algorithm (UMAP+HDBSCAN), six pharmacogenetic clusters were defined based on the genotypes of 28 genes. Due to the geometric nature of the clusters in a two-dimensional space, density-based clustering was more suitable compared to means-based clustering. Each cluster has specific genotypes by which it can be identified, predominantly in the *CYP2D6* gene. Phenotype analysis revealed cluster-based variance in 14 out of 28 genes, providing cluster-specific phenotypes for those genes.

### 3.1. CYP Family Cluster-Specific Phenotype Considerations

Genes belonging to the *CYP* family that showed phenotype cluster variability included *CYP1A2, CYP2B6, CYP2C9, CYP2D6, CYP3A4,* and *CYP3A5*.

Regarding *CYP1A2*, patients in clusters 1, 3, and 4 did not possess the intermediate to normal phenotype, which is associated with a lower enzyme activity and drug clearance rate. *CYP1A2* is involved in the metabolism of several clinically relevant drugs, such as imipramine, clozapine, theophylline, and mexiletine, as well as caffeine and some procarcinogens [11]. Patients with lower clearance rates might be more prone to adverse reactions with these drugs, which include serious side effects. Toxic effects of imipramine include anticholinergic action, which can lead to tachycardia, constipation, or glaucoma, as well as serotonin syndrome, a life-threatening condition [12]. Clozapine can cause agranulocytosis, myocarditis, or seizures [13]. Mexiletine toxicity has been associated with arrhythmias, hypotension, altered mental status, ataxia and paresthesia, esophagitis, etc., ref. [14].

*CYP2B6* demonstrated higher phenotype variability than *CYP1A2*. Cluster 1 did not contain poor metabolizers, cluster 2 did not contain ultrarapid metabolizers, cluster 3 did not contain poor, intermediate to normal, or ultrarapid metabolizers, and clusters 4, 5, and 6 did not contain intermediate to normal or ultrarapid metabolizers. Patients with poor or intermediate to normal metabolism have lower enzyme activity, while ultrarapid metabolizers have higher enzyme activity, making them more prone to therapeutic ineffectiveness. *CYP2B6* is involved in the metabolism of cyclophosphamide, efavirenz, bupropion, and ifosfamide, among others [11]. Therapeutic ineffectiveness in the case of cyclophosphamide and ifosfamide can cause reduced antineoplastic activity and reduce cancer treatment success. On the other hand, slower clearance of these drugs can cause adverse effects in multiple organ systems, some of which include encephalopathy, hematuria, liver injury, arrhythmia, etc., [15,16]. Bupropion can exhibit a lesser antidepressant effect in faster metabolizers and multiple organ system side effects in slower metabolizers, including lowering seizure thresholds and worsening suicide tendencies [17].

Regarding *CYP2C9*, poor metabolizers were not found in clusters 1, 3, 4, and 6. *CYP2C9* is involved in the metabolism of many drugs, like phenytoin, non-steroidal anti-inflammatory drugs, and warfarin [18]. Poor *CYP2C9* metabolizers taking warfarin may be at increased risk of hemorrhage-related side effects which can potentially be life-threatening [19]. Poor metabolizers taking NSAIDs can be at increased risk of drug-induced toxicity (gastro-, cardio- and nephrotoxicity) [20]. Phenytoin toxicity is associated with a wide range of adverse reactions, including specific syndromes such as Stevens–Johnson syndrome, Dress syndrome, Purple glove syndrome, and other conditions [21].

*CYP2D6* also showed greater phenotype variability among clusters. Poor and poor to intermediate metabolizers were not present in clusters 1, 2, and 3. Additionally poor metabolizers were also absent from cluster 5. Cluster 3 also did not include any intermediate to normal or intermediate metabolizers. As for higher enzyme activity, cluster 1 did not contain ultrarapid metabolizers, while clusters 2, 3, 4 and 5 did not contain rapid metabolizers. *CYP2D6* is responsible for metabolizing up to 30% of drugs, including some antineoplastic drugs, opioid analgesics, antiemetic drugs, antidepressants (tricyclic and SSRIs), antiarrhythmics, beta-blockers, neuroleptics, etc., [22]. Codeine, an example of opioid analgesics, is converted by *CYP2D6* to morphine, a much more potent substance. Rapid and ultrarapid metabolizers can exhibit much higher morphine levels after codeine administration, resulting in adverse effects similar to those of an opioid overdose. Moreover, opioid-related newborn deaths have been reported in breastfeeding mothers who were treated with codeine and were ultrarapid *CYP2D6* metabolizers. On the other hand, individuals with weaker *CYP2D6* activity experience ineffectiveness where analgesia is concerned [23]. Altered *CYP2D6* metabolism of antidepressants, antineoplastics, and antiarrhythmics results in the previously discussed effects for these groups of drugs.

A normal *CYP3A4* metabolic rate was present in all clusters. On the other hand, an intermediate metabolic rate was present only in cluster 3 and absent in the rest. Regarding *CYP3A5*, poor and intermediate metabolizers were present in all clusters. However, normal metabolizers were specific to cluster 5. The CYP3A subfamily is also responsible for a large portion of drug metabolism, including oncology-related drugs such as cyclophosphamide, ifosfamide, irinotecan, tyrosine kinase inhibitors, etoposide, docetaxel, tamoxifen, etc. [24]. It has been shown that reduced systemic clearance of cyclophosphamide can result in a significantly poorer response to treatment [25].

### 3.2. HLA Cluster-Specific Phenotype Considerations

Regarding the *HLA-A* locus, all clusters included the negative genotype associated with normal risk. However, the *31:01 genotype associated with increased risk was specific to cluster 6 and not present in the other five clusters. The significance of this *HLA-A* genotype has been investigated. McCormack et al. (2011), investigating the European population, found it to be significantly associated with carbamazepine-induced adverse effects, such as maculopapular exanthema, hypersensitivity syndrome, and Stevens–Johnson syndrome with toxic epidermal necrolysis [26]. Ozeki et al. (2011) arrived at a similar conclusion while conducting their study on the Japanese population [27]. Furthermore, it is important to emphasize that long-term observational therapeutic drug monitoring studies on carbamazepine have highlighted the significance of pharmacogenetic testing in optimizing therapeutic outcomes. Grzesk et al. (2021) re-ported the results of their 20-year observational study, which revealed that only 71% of the 710 patients taking carbamazepine achieved serum drug concentrations within the therapeutic range. Meanwhile, 24.9% had concentrations below the therapeutic level, and 4.1% had concentrations exceeding it [7]. These findings further underscore the importance of a personalized therapeutic approach to ensure optimal drug efficacy and safety.

In terms of the *HLA-B* locus, a normal-risk genotype was found, as were two increased-risk genotypes. The *57:01 genotype is associated with an increased risk with abacavir and pazopanib, while the *58:01 genotype is associated with an increased risk with allopurinol. The normal risk and *57:01 patients were distributed among all clusters. The *58:01 genotype was found in all clusters except cluster 4. The risk associated with *58:01 and allopurinol therapy refers to an increased incidence of severe cutaneous adverse reactions (SCARs) [28].

### 3.3. Cluster-Specific Phenotype Considerations of Other Genes

Further phenotype variance was found in the *DPYD* gene. While the normal-risk phenotype was distributed among all clusters, clusters 2 and 5 did not contain the increased-risk (DPD score 1.5) phenotype and cluster 3 did not contain the increased-risk (DPD score 1) phenotype. Furthermore, cluster 4 did not contain either of the increased-risk phenotypes, but rather only patients with normal risk. *DPYD* plays a key role in the metabolism of fluoropyrimidines, such as 5-fluorouracil, a commonly used antineoplastic agent. The enzyme activity is objectivized by the DPD score, with a value of 2 representing normal activity and lower values representing lesser activity. Genetic DPD deficiencies are associated with 5-fluorouracil toxicity [29]. For this reason, guidelines have been published to guide 5-fluorouracil doses based on *DPYD* genetic findings [30].

Regarding *NUDT15*, normal metabolizers were found in all clusters. However, increased-risk individuals were not present in clusters 2, 3, and 4. It has been demonstrated that children suffering from acute lymphoblastic leukemia treated with 6-mercaptopurine have an increased risk of induced neutropenia if the *NUDT15* rs116855232 C/rs116855232 T genotype is present [31]. Additionally, it has been demonstrated that children with *NUDT15* polymorphisms suffering from acute lymphoblastic leukemia have a higher risk of second malignancies [32].

The *OPRM1* gene Asn/Asn and Asn/Asp isoforms were present in all clusters. The Asp/Asp isoform was present in clusters 1, 5, and 6 and absent from clusters 2, 3, and 4. The *OPRM1* gene encodes the μ opioid receptor, the most important component of the effector path of opioid analgesics. It has been reported that the *OPRM1* Asn40Asp polymorphism impacts an individual’s response to opioid receptor antagonists naloxone and naltrexone, and can serve as a prognostic factor for recovering alcoholics [33]. Additionally, the OPRM1-A118G polymorphism has shown an association with a reduced risk of postoperative vomiting [34].

Greater phenotype variability was also detected in the *SLCO1B1* gene. Cluster 3 did not include the poor-function and decreased-function groups. The poor-function group was also absent from cluster 6, alongside the reduced-response and normal-risk group. Finally, the decreased-function group was also not present in clusters 1, 2, and 4. *SLCO1B1* has been demonstrated as a significant transporter for endogenous substrates, such as hormones and bilirubin. *SLCO1B1* polymorphisms may also negatively interfere with certain chemotherapeutic agents such as methotrexate, resulting in poorer treatment outcomes [35]. Additionally, it has also been demonstrated that *SLCO1B1* polymorphisms, particularly the *5/*5 genotype, may be associated with an increased risk for statin-induced myopathy and hyperbilirubinemia [36].

Distributions of the low-activity, intermediate-activity, and normal-activity phenotypes of the *VKORC1* gene included all six clusters. However, the resistance allele phenotype was specific to cluster 1, and absent from clusters 2 to 6. The resistance genotype pertains to warfarin resistance and may lead to the therapeutic ineffectiveness of warfarin therapy [37]. This can lead to a failure of anticoagulant effect to take place and thromboembolic incidents.

Finally, cluster 1 did not contain any patients with an *F5* increased-risk phenotype, while the normal-risk phenotype was present in all clusters. The *F5* rs6025 A polymorphism makes the F5 protein more difficult to cleave by activated protein C. This makes the individual with the increased-risk phenotype more prone to venous thrombosis and thromboembolic events [38].

### 3.4. Limitations

While the results of this study provided some interesting insight into the pharmacogenomic composition of the Croatian population, its limitations must be stated. The main limitation of this study is the sample size. While the clustering analysis yielded promising results, a larger dataset is essential to validate contributing factors, minimize noise, and enhance patient classification accuracy. Further research is needed to gather a sufficiently large sample for robust machine learning analysis and the development of clinically applicable guidelines. If pharmacogenetic clustering was conducted on a larger cohort of patients, the phamacogenomic map would likely have a different spatial composition of clusters and subsequently different genotype/phenotype distributions. It is only through a large-enough population sample that we can be sufficiently confident in the discovered associations to suggest the clinical implementation of a pharmacogenetics-based classification system. Additionally, another limitation is the methodologies used, namely PCR probe-based methods, which can only detect specific gene variants. With the increasing adoption of next-generation sequencing (NGS) technologies, future studies will benefit from larger datasets and the more extensive detection of gene variants associated with pharmacogenetic effects. Our future research will focus on whole-genome sequencing (WGS) to generate more comprehensive data, which will be analyzed using machine learning techniques to develop more accurate pharmacogenetic clusters for clinical application.

Secondly, this pharmacogenomic map pertains only to the Croatian population. As cluster–phenotype associations for other populations may be different for other populations, this knowledge may not be applicable to these. It is worth noting, however, that our previous pharmacogenomic population study demonstrated that no significant differences were present between the Croatian and European populations, regarding genes investigated in this study [2].

Thirdly, as dimensionality reduction was applied prior to clustering, there is a certain aspect of ambiguity that must be taken into account in regard to multidimensional density and distance preservation. This is currently a topic of debate on various programming-related forums, as some state that clustering on dimensionality reduction results can yield a disingenuous clustering pattern. However, using UMAP prior to density-based clustering has been stated as a valid method in the UMAP documentation [39]. Furthermore, the validation of clustering was performed by predictive modeling based on the original multidimensional database, not the reduced UMAP components, and yielded excellent results (avg. ROC-AUC = 0.998).

### 3.5. Conclusions

This study demonstrated pharmacogenetic clustering of the Croatian population using an UMAP + HDBSCAN machine learning algorithm. CYP2D6 and SLCO1B1 genotypes turned out to be highly specific between clusters, allowing highly accurate cluster identification based on these two genes. Each cluster provided specific and clinically relevant knowledge regarding pharmacogenetic phenotypes, mainly by excluding certain ones. With the issue of the sample size thoroughly discussed in the limitations section, it is clear that further research on larger patient cohorts in this direction would be prudent for the creation of any clinically applicable classification system. With larger volumes of pharmacogenetic data and robust machine learning algorithms used to analyze them, novel associations between significant genotypes and phenotypes might be discovered, positively impacting the evolvement of personalized treatment guidelines.

## 4. Materials and Methods

### 4.1. Data Curation

The data used in this article consisted of individual pharmacogenetic testing results obtained retrospectively from the St. Catherine Specialty Hospital clinical archive. The data were extracted with approval from the institutional ethics committee of St. Catherine Specialty Hospital (September 2024, 24/9-I). The data used for this machine learning-based analysis were primarily obtained through PCR probe-based methods, which are thoroughly characterized and compared with European populations in our previous population studies [2,6]. The pharmacogenetic testing findings available in our archive comprised 28 genes, including *CYP1A2, CYP2B6, CYP2C9, CYP2C19, CYP2C cluster, CYP2D6, CYP3A4, CYP3A5, CYP4F2, COMT, DPYD, DRD2, GRIK4, HLA-A, HLA-B, HTR2A, HTR2C, IFNL4, NUDT15, OPRM1, SLC6A4, SLCO1B1, TPMT, UGT1A1, VKORC1, F II, F V,* and *MTHFR.* For each gene, the exact genotype and its corresponding phenotype were available in each entry. All genes and all entries available in the archive were utilized in this study, producing a database of 522 entries and 58 features.

### 4.2. Data Preprocessing

Data preprocessing involved the numerical encoding of categorical allele variants for each gene, the creation of a secondary database serving as a dictionary for numerical encoding, and the creation of a secondary database serving as a dictionary for genotype-phenotype correlations. Each gene corresponded to two columns in the database, one for each allele. The exceptions were *HLA-A* and *HLA-B,* which had only one corresponding column each, and *SLCO1B1* and *MTHFR*, which had four corresponding columns each. *MTHFR* analysis involved two separate polymorphisms, each of which had two corresponding alleles per patient. *SLCO1B1*, in some cases, had “uncertain” genotypes, which comprised two genotypes (4 alleles) that the test could not differentiate with certainty.

### 4.3. General Programming Language and Environment Details

The data processing in this study was performed in the Python programming language (v3.12.3) using several Python libraries (Pandas v2.2.2, NumPy v1.26.0, MatPlotLib v3.8.0, SciKit-Learn v1.5.1, XGBoost v2.1.0, and UMAP-Learn v0.5.6). The code was executed in the form of a Jupyter Notebook, in the Visual Studio Code software.

### 4.4. Cluster Determination

For patient clustering, we employed unsupervised machine learning algorithms. The first step involved dimensionality reduction using the Uniform Manifold Approximation and Projection (UMAP) algorithm. Hyperparameters “n_components” and “random_state” were set to 2 and 42, respectively, while the rest were left as default. The dimensions of the initial database were reduced to two components which served as the basis for clustering. The second step was the clustering itself, which was performed using the Hierarchical Density-Based Spatial Clustering of Applications with Noise (HDBSCAN) algorithm. The “min_cluster_size” hyperparameter was set to 30, while the rest were left as default. Unsuccessfully clustered samples (identified as noise and labeled as -1 by HDBSCAN) were removed from further analysis. The silhouette score was determined as a form of clustering validation.

### 4.5. Cluster Analysis

Once the clustering was finished, the original database was divided based on the determined cluster labels. Further processing involved the allele decoding and identification of unique genotypes present in each cluster for each gene and the determination of the presence of each genotype for each cluster. Further genotype cluster analysis involved the determination of positively specific genotypes (present in only one cluster) and negatively specific genotypes (not present in only one cluster). Using the corresponding genotype–phenotype database, the presence of each phenotype in each cluster was also determined. Similarly to genotypes, positively specific and negatively specific phenotypes were determined for each cluster. Finally, each cluster was characterized by the phenotypes it contained.

### 4.6. Cluster Validation

Validation of the clustering process was performed using supervised machine learning predictive models. All models were initialized with default hyperparameters and random states set to 42 to ensure reproducibility. A simple DecisionTreeClassifier was fitted on the whole database in order to produce a visualized and interpretable decision tree and discern which genes and alleles were most important in cluster determination. Following this, prediction quality testing was performed. The input data were from the initial database processed by the UMAP algorithm and the output classes were the labels assigned by clustering. The data were split into separate subsets used for model training and model testing, using a 70:30 train–test split stratified by output class. Three ensemble tree-based models were trained on the data, including the RandomForestClassifier, XGBoostClassifier, and ExtraTreesClassifier. Prediction quality was assessed by the accuracy score, F1 score (weighted), receiver operating characteristic (ROC) curve, and area under ROC curve (ROC-AUC) score (one-versus-rest, weighted). Means based on all three models were calculated for each metric.

## Figures and Tables

**Table 1:** Positively and negatively specific genotypes across genes and clusters.

Gene	Positively Specific Genotypes	Negatively Specific Genotypes
*CYP2B6*	CL1: *4/*4	
*CYP2C9*		CL2: *2/*2
*CYP2D6*	CL1: *1/*14, *1/*41x3, *1x2/*41 CL2: *1/*2Ax2, *1/*6, *1x2/*2Ax2, *1x2/*4 CL3: *1/*1, *1/*1x2 CL4: *2+*13/*4, *2A/*10, *2A/*4, *2A/*4+*4N, *2A/*4x2, *2A/*9, *2Ax2/*4+*68, *3/*4+*68, *3/*4+*68xN, *3/*5, *4+*68/*4+*68, *4/*10, *4/*5, *5/*5 CL5: *10/*41, *13/*39, *2A+13/*35, *2A+13/*41, *2A/*13+*2A, *2A/*59, *2Ax2/*41, *35/*41, *35/*59, *4+*4N/*41, *41/*41, *41x2/*59, *4x2/*35, *5/*35, *5/*41, *59/*59, *6/*41, *9/*13, *9/*35, *9/*41 CL6: *1/*2x2, *2/*4, *2Ax2/*4, *2Ax2/*4+*68xN, *2X2/*41, *3/*35, *4+*4N/*9, *4+*68/*5, *4/*59, *4x2/*4+*4N	
*CYP3A4*	CL3: *22/*22	
*CYP3A5*	CL3: *3/*7 CL5: *1/*1	
*HLA-A*	CL6: Positive *31:01	
*HLA-B*		CL4: Positive *58:01
*SLCO1B1*	CL1: *21/*21 CL2: *17/*17 CL5: *5/*17 CL6: *1/*17/*5/*21, *1A/*17/*5/*21	CL6: *1A/*1A, *1A/*1B, *1A/*21, *1A/*5, *1B/*15, *1B/*1B
*VKORC1*	CL1: rs9923231 G/G resistance allele/rs9923231 G/G resistance allele	
*F5*		CL1: rs6025 G/rs6025 A

CLx—Cluster x.

**Table 2:** Positively and negatively specific phenotypes across genes and clusters.

Gene	Positively Specific Phenotypes	Negatively Specific Phenotypes
*CYP2B6*	CL1: ultrarapid	
*CYP2D6*		CL1: ultrarapid CL3: intermediate, intermediate to normal
*CYP3A4*	CL3: intermediate	
*CYP3A5*	CL5: normal	
*HLA-A*	CL6: increased risk	
*HLA-B*		CL4: increased risk with allopurinol
*SLCO1B1*		CL6: reduced response, normal risk
*VKORC1*	CL1: resistance allele	
*F5*		CL1: increased risk

CLx—Cluster x.

**Table 3:** Quality metrics of predictive model cluster validation.

Model	Metric	All Genes	*CYP2D6* *+ SLCO1B1*	*CYP2D6*	*SLCO1B1*
RandomForestClassifier	Accuracy	0.993464	1.000000	0.816993	0.385621
	F1	0.901961	1.000000	0.769094	0.347213
	ROC-AUC	0.999923	1.000000	0.911309	0.660545
XGBoostClassifier	Accuracy	0.993464	0.993464	0.816993	0.372549
	F1	0.993419	0.993419	0.776162	0.341668
	ROC-AUC	1.000000	1.000000	0.911771	0.664012
ExtraTreesClassifier	Accuracy	0.901961	1.000000	0.810458	0.379085
	F1	0.900774	1.000000	0.748300	0.363386
	ROC-AUC	0.993114	1.000000	0.908297	0.660164
AVERAGE	Accuracy	0.962963	0.997821	0.814815	0.379085
	F1	0.962549	0.997806	0.764519	0.350756
	ROC-AUC	0.997679	1.000000	0.910459	0.661574

## Data Availability

All of the research data are included in the manuscript.

## Supplementary Materials

The following supporting information can be downloaded at https://www.mdpi.com/article/10.3390/ijms26020589/s1.
