# Supplementary material for: Exploring the Pharmacogenomic Map of Croatia: PGx Clustering of 522-Patient Cohort Based on UMAP + HDBSCAN Algorithm
Source: Int J Mol Sci. 2025 Jan 12;26(2):589. doi: 10.3390/ijms26020589 (PMC11765990; doi:10.3390/ijms26020589)
Supplement: Supplementary file 1 [file ijms-26-00589-s001.zip › Supplement table S1.pdf]

Supplement Table S1. Distribution of each genotype among clusters

Gene	Genotype	1	2	3	4	5	6
CYP1A2	*1A/*1A	+	+	+	+	+	+
	*1A/*1F	+	+	+	+	+	+
	*1A/*1L	+	+	-	+	-	+
	*1D/*1F	+	-	+	+	+	-
	*1D/*1J	-	+	+	+	-	+
	*1F/*1F	+	+	+	+	+	+
	*1F/*1L	-	+	+	+	+	-
	*1F/*1V	-	+	-	+	+	+
	*1F/*1W	+	+	+	+	+	+
	*1K/*1V	-	+	-	-	+	+
CYP2B6	*1/*1	+	+	+	+	+	+
	*1/*4	+	+	+	+	+	+
	*1/*5	+	+	+	+	+	+
	*1/*6	+	+	+	+	+	+
	*4/*4	+	-	-	-	-	-
	*4/*5	-	-	+	-	+	-
	*4/*6	+	+	-	-	-	-
	*5/*5	-	+	-	+	+	+
	*5/*6	+	+	+	+	+	+
	*6/*6	+	+	+	+	+	+
CYP2C9	*1/*1	+	+	+	+	+	+
	*1/*11	-	-	+	+	-	-
	*1/*2	+	+	+	+	+	+
	*1/*3	+	+	+	+	+	+
	*2/*2	+	-	+	+	+	+
	*2/*3	+	+	+	+	+	+
	*3/*3	-	+	-	-	+	-
CYP2C19	*1/*1	+	+	+	+	+	+
	*1/*17	+	+	+	+	+	+
	*1/*2	+	+	+	+	+	+
	*17/*17	+	+	+	+	+	+
	*2/*17	+	+	+	+	+	+
	*2/*2	+	+	+	+	+	+
CYP2C-cluster	rs12777823 A/rs12777823 A	+	+	+	+	+	+
	rs12777823 G/rs12777823 A	+	+	+	+	+	+
	rs12777823 G/rs12777823 G	+	+	+	+	+	+
CYP2D6	*1/*1	-	-	+	-	-	-
	*1/*10	-	+	-	-	-	+
	*1/*14	+	-	-	-	-	-
	*1/*1x2	-	-	+	-	-	-
	*1/*2A	-	+	-	-	-	+
	*1/*2A+*13	-	+	-	-	-	+
	*1/*2Ax2	-	+	-	-	-	-
	*1/*2x2	-	-	-	-	-	+
	*1/*3	-	+	-	-	-	+

*1/*35	+	-	-	-	-	+
*1/*4	-	+	-	-	-	+
*1/*4+*4N	-	+	-	-	-	+
*1/*4+*68	-	+	-	-	-	+
*1/*41	+	-	-	-	-	+
*1/*41x3	+	-	-	-	-	-
*1/*5	-	+	-	-	-	+
*1/*59	+	-	-	-	-	+
*1/*6	-	+	-	-	-	-
*1/*9	-	+	-	-	-	+
*10/*41	-	-	-	-	+	-
*13/*39	-	-	-	-	+	-
*1x2/*2A	-	+	-	-	-	+
*1x2/*2Ax2	-	+	-	-	-	-
*1x2/*4	-	+	-	-	-	-
*1x2/*41	+	-	-	-	-	-
*2+*13/*4	-	-	-	+	-	-
*2/*4	-	-	-	-	-	+
*2A+*13/*35	-	-	-	-	+	-
*2A+*13/*41	-	-	-	-	+	-
*2A/*10	-	-	-	+	-	-
*2A/*13+*2A	-	-	-	-	+	-
*2A/*2A	-	-	-	+	-	+
*2A/*2Ax2	-	-	-	+	-	+
*2A/*35	-	-	-	-	+	+
*2A/*4	-	-	-	+	-	-
*2A/*4+*4N	-	-	-	+	-	-
*2A/*4+*68	-	-	-	+	-	+
*2A/*41	-	-	-	-	+	+
*2A/*4x2	-	-	-	+	-	-
*2A/*5	-	-	-	+	-	+
*2A/*59	-	-	-	-	+	-
*2A/*6	-	-	-	+	-	+
*2A/*9	-	-	-	+	-	-
*2Ax2/*4	-	-	-	-	-	+
*2Ax2/*4+*68	-	-	-	+	-	-
*2Ax2/*4+*68xN	-	-	-	-	-	+
*2Ax2/*41	-	-	-	-	+	-
*2x2/*41	-	-	-	-	-	+
*3/*35	-	-	-	-	-	+
*3/*4	-	-	-	+	-	+
*3/*4+*68	-	-	-	+	-	-
*3/*4+*68xN	-	-	-	+	-	-
*3/*5	-	-	-	+	-	-
*35/*41	-	-	-	-	+	-
*35/*59	-	-	-	-	+	-
*4+*4N/*35	-	-	-	-	+	+
*4+*4N/*41	-	-	-	-	+	-

	*4+*4N/*9	-	-	-	-	-	+
	*4+*68/*35	-	-	-	-	+	+
	*4+*68/*4+*68	-	-	-	+	-	-
	*4+*68/*41	-	-	-	-	+	+
	*4+*68/*5	-	-	-	-	-	+
	*4/*10	-	-	-	+	-	-
	*4/*35	-	-	-	-	+	+
	*4/*4	-	-	-	+	-	+
	*4/*4+*68	-	-	-	+	-	+
	*4/*41	-	-	-	-	+	+
	*4/*5	-	-	-	+	-	-
	*4/*59	-	-	-	-	-	+
	*4/*9	-	-	-	+	-	+
	*41/*41	-	-	-	-	+	-
	*41x2/*59	-	-	-	-	+	-
	*4x2/*35	-	-	-	-	+	-
	*4x2/*4+*4N	-	-	-	-	-	+
	*5/*35	-	-	-	-	+	-
	*5/*41	-	-	-	-	+	-
	*5/*5	-	-	-	+	-	-
	*59/*59	-	-	-	-	+	-
	*6/*41	-	-	-	-	+	-
	*9/*13	-	-	-	-	+	-
	*9/*35	-	-	-	-	+	-
	*9/*41	-	-	-	-	+	-
CYP3A4	*1/*1	+	+	+	+	+	+
	*1/*1B	+	+	+	+	+	+
	*1/*22	+	+	+	+	+	+
	*22/*22	-	-	+	-	-	-
CYP3A5	*1/*1	-	-	-	-	+	-
	*1/*3	+	+	+	+	+	+
	*3/*3	+	+	+	+	+	+
	*3/*7	-	-	+	-	-	-
CYP4F2	*1/*1	+	+	+	+	+	+
	*1/*3	+	+	+	+	+	+
	*3/*3	+	+	+	+	+	+
COMT	rs4680 A/rs4680 A	+	+	+	+	+	+
	rs4680 G/rs4680 A	+	+	+	+	+	+
	rs4680 G/rs4680 G	+	+	+	+	+	+
DPYD	*1/*1	+	+	+	+	+	+
	*1/*2A	+	+	-	-	+	+
	rs67376798 T/rs67376798 A	+	-	+	-	-	+
DRD2	rs1799978 A/rs1799978 A	+	+	+	+	+	+
	rs1799978 A/rs1799978 G	+	+	+	+	+	+
GRIK4	rs1954787 C/rs1954787 C	+	+	+	+	+	+
	rs1954787 T/rs1954787 C	+	+	+	+	+	+
	rs1954787 T/rs1954787 T	+	+	+	+	+	+
HLA-A	Negative	+	+	+	+	+	+

	Positive *31:01	-	-	-	-	-	+
HLA-B	Negative	+	+	+	+	+	+
	Positive *57:01	+	+	+	+	+	+
	Positive *58:01	+	+	+	-	+	+
HTR2A	rs7997012 A/rs7997012 A	+	+	+	+	+	+
	rs7997012 A/rs7997012 G	+	+	+	+	+	+
	rs7997012 G/rs7997012 G	+	+	+	+	+	+
HTR2C	rs3813929 C/rs3813929 C	+	+	+	+	+	+
	rs3813929 C/rs3813929 T	+	+	+	+	+	+
	rs3813929 T/rs3813929 T	+	+	+	+	+	+
IFNL4	rs12979860 C/rs12979860 C	+	+	+	+	+	+
	rs12979860 C/rs12979860 T	+	+	+	+	+	+
	rs12979860 T/rs12979860 T	+	+	+	+	+	+
NUDT15	rs116855232 C/rs116855232 C	+	+	+	+	+	+
	rs116855232 C/rs116855232 T	+	-	-	-	+	+
OPRM1	rs1799971 A/rs1799971 A	+	+	+	+	+	+
	rs1799971 A/rs1799971 G	+	+	+	+	+	+
	rs1799971 G/rs1799971 G	+	-	-	-	+	+
SLC6A4	La/La	+	+	+	+	+	+
	La/Lg	+	+	+	+	+	+
	La/Sa	+	+	+	+	+	+
	Lg/Sa	+	+	+	+	+	+
	Sa/Sa	+	+	+	+	+	+
SLCO1B1	*1/*17/*5/*21	-	-	-	-	-	+
	*1/*21/#/#	-	+	-	+	+	-
	*15/*15/#/#	+	+	-	+	+	-
	*15/*21/*1B/*17	-	-	-	-	+	+
	*17/*17/#/#	-	+	-	-	-	-
	*17/*21/#/#	+	-	-	-	+	-
	*1A/*15/*1B/*5	-	-	-	-	+	+
	*1A/*17/*5/*21	-	-	-	-	-	+
	*1A/*1A/#/#	+	+	+	+	+	-
	*1A/*1B/#/#	+	+	+	+	+	-
	*1A/*21/#/#	+	+	+	+	+	-
	*1A/*5/#/#	+	+	+	+	+	-
	*1B/*15/#/#	+	+	+	+	+	-
	*1B/*1B/#/#	+	+	+	+	+	-
	*1B/*21/#/#	+	+	-	-	-	-
	*21/*21/#/#	+	-	-	-	-	-
	*5/*15/#/#	+	+	-	-	-	-
	*5/*17/#/#	-	-	-	-	+	-
	*5/*5/#/#	-	+	-	+	-	-
TPMT	*1/*1	+	+	+	+	+	+
	*1/*3A	+	+	+	+	+	+
	*1/*3C	+	+	-	-	-	-
UGT1A1	*1/*1	+	+	+	+	+	+
	*1/*28	+	+	+	+	+	+
	*1/*6	-	+	-	-	+	-

	*28/*28	+	+	+	+	+	+
	*6/*28	-	+	-	-	-	+
VKORC1	rs9923231 A/rs9923231 A	+	+	+	+	+	+
	rs9923231 G/G resistance allele/rs9923231 G/G resistance allele	+	-	-	-	-	-
	rs9923231 G/rs9923231 A	+	+	+	+	+	+
	rs9923231 G/rs9923231 G	+	+	+	+	+	+
F2	rs1799963 G/rs1799963 A	+	+	+	+	+	+
	rs1799963 G/rs1799963 G	+	+	+	+	+	+
F5	rs6025 G/rs6025 A	-	+	+	+	+	+
	rs6025 G/rs6025 G	+	+	+	+	+	+
MTHFR	rs1801133 C/rs1801133 C/rs1801131 A/rs1801131 A	+	+	+	+	+	+
	rs1801133 C/rs1801133 C/rs1801131 A/rs1801131 C	+	+	+	+	+	+
	rs1801133 C/rs1801133 C/rs1801131 C/rs1801131 C	+	+	+	+	+	+
	rs1801133 C/rs1801133 T/rs1801131 A/rs1801131 A	+	+	+	+	+	+
	rs1801133 C/rs1801133 T/rs1801131 A/rs1801131 C	+	+	+	+	+	+
	rs1801133 T/rs1801133 T/rs1801131 A/rs1801131 A	+	+	+	+	+	+

Negatively specific genotypes; Positively specific genotypes; Completely non-specific genes; + present; - absent; # - no alternative allele
