# Supplementary material for: Exploring the Pharmacogenomic Map of Croatia: PGx Clustering of 522-Patient Cohort Based on UMAP + HDBSCAN Algorithm
Source: Int J Mol Sci. 2025 Jan 12;26(2):589. doi: 10.3390/ijms26020589 (PMC11765990; doi:10.3390/ijms26020589)
Supplement: Supplementary file 1 [file ijms-26-00589-s001.zip › Supplement table S2.pdf]

Supplement Table S2. Distribution of each phenotype among clusters

Gene	Phenotype	1	2	3	4	5	6
CYP1A2	intermediate to normal	-	+	-	-	+	+
	normal	+	+	+	+	+	+
	rapid	+	+	+	+	+	+
CYP2B6	poor	-	+	-	+	+	+
	poor to intermediate	+	+	+	+	+	+
	intermediate	+	+	+	+	+	+
	intermediate to normal	+	+	-	-	-	-
	normal	+	+	+	+	+	+
	rapid	+	+	+	+	+	+
	ultrarapid	+	-	-	-	-	-
CYP2C9	poor	-	+	-	-	+	-
	poor to intermediate	+	+	+	+	+	+
	intermediate	+	+	+	+	+	+
	intermediate to normal	+	+	+	+	+	+
	normal	+	+	+	+	+	+
CYP2C19	poor	+	+	+	+	+	+
	intermediate	+	+	+	+	+	+
	intermediate to normal	+	+	+	+	+	+
	normal	+	+	+	+	+	+
	rapid	+	+	+	+	+	+
	ultrarapid	+	+	+	+	+	+
CYP2C-cluster	normal	+	+	+	+	+	+
	variant present	+	+	+	+	+	+
CYP2D6	poor	-	-	-	+	-	+
	poor to intermediate	-	-	-	+	+	+
	intermediate	+	+	-	+	+	+
	intermediate to normal	+	+	-	+	+	+
	normal	+	+	+	+	+	+
	rapid	+	-	-	-	-	+
	ultrarapid	-	+	+	+	+	+
CYP3A4	intermediate	-	-	+	-	-	-
	normal	+	+	+	+	+	+
CYP3A5	poor	+	+	+	+	+	+
	intermediate	+	+	+	+	+	+
	normal	-	-	-	-	+	-
CYP4F2	normal	+	+	+	+	+	+
	reduced activity	+	+	+	+	+	+
COMT	low activity	+	+	+	+	+	+
	intermediate activity	+	+	+	+	+	+
	high activity	+	+	+	+	+	+
DPYD	normal risk	+	+	+	+	+	+
	increased risk (DPD score = 1)	+	+	-	-	+	+
	increased risk (DPD score = 1.5)	+	-	+	-	-	+
DRD2	normal response	+	+	+	+	+	+
	reduced response	+	+	+	+	+	+

GRIK4	normal response	+	+	+	+	+	+
	risk of reduced response	+	+	+	+	+	+
HLA-A	normal risk	+	+	+	+	+	+
	increased risk	-	-	-	-	-	+
HLA-B	normal risk	+	+	+	+	+	+
	increased risk with abacavir and pazopanib	+	+	+	+	+	+
	increased risk with allopurinol	+	+	+	-	+	+
HTR2A	intron 2 genotype AA	+	+	+	+	+	+
	intron 2 genotype AG	+	+	+	+	+	+
	intron 2 genotype GG	+	+	+	+	+	+
HTR2C	normal risk	+	+	+	+	+	+
	protective effect	+	+	+	+	+	+
IFNL4	normal response	+	+	+	+	+	+
	reduced response	+	+	+	+	+	+
	reduced response	+	+	+	+	+	+
NUDT15	normal metabolizer	+	+	+	+	+	+
	increased risk	+	-	-	-	+	+
OPRM1	Asn/Asn isoform	+	+	+	+	+	+
	Asn/Asp isoform	+	+	+	+	+	+
	Asp/Asp isoform	+	-	-	-	+	+
SLC6A4	typical to increased expression	+	+	+	+	+	+
	likely typical to reduced expression	+	+	+	+	+	+
	typical to reduced expression	+	+	+	+	+	+
	likely reduced expression	+	+	+	+	+	+
	reduced expression	+	+	+	+	+	+
SLCO1B1	poor function	+	+	-	+	+	-
	decreased function	-	-	-	-	+	+
	reduced response	+	+	+	+	+	-
	normal risk	+	+	+	+	+	-
	increased risk	+	+	+	+	+	+
TPMT	normal risk	+	+	+	+	+	+
	increased risk	+	+	+	+	+	+
UGT1A1	normal risk	+	+	+	+	+	+
	increased risk	+	+	+	+	+	+
	high risk	+	+	+	+	+	+
VKORC1	low activity	+	+	+	+	+	+
	resistance allele	+	-	-	-	-	-
	intermediate activity	+	+	+	+	+	+
	normal activity	+	+	+	+	+	+
F2	increased risk	+	+	+	+	+	+
	normal risk	+	+	+	+	+	+
F5	increased risk	-	+	+	+	+	+
	normal risk	+	+	+	+	+	+
MTHFR	normal activity	+	+	+	+	+	+
	decreased activity	+	+	+	+	+	+
	severely decreased activity	+	+	+	+	+	+

Negatively specific phenotypes; Positively specific phenotypes; Completely non-specific genes; + present; - absent
